# Indications and Short-Term Outcomes for In-Office Therapeutic Superior Laryngeal Nerve Block

**DOI:** 10.1177/00034894231194384

**Published:** 2023-08-22

**Authors:** Alan J. Gray, Matthew R. Hoffman, Zao M. Yang, Beau Vandiver, Joshua Purvis, Jake P. Morgan, Edie R. Hapner, Laura Dominguez, Kathleen Tibbetts, C. Blake Simpson

**Affiliations:** 1 Department of Otolaryngology—Head and Neck Surgery, University of Texas Health San Antonio, San Antonio, TX, USA; 2 Department of Otolaryngology—Head and Neck Surgery, University of Alabama-Birmingham, Birmingham, AL, USA; 3 Department of Otolaryngology—Head and Neck Surgery, University of Iowa, Iowa City, IA, USA; 4 Department of Otolaryngology—Head and Neck Surgery, Cleveland Clinic Florida, Coral Springs, FL, USA; 5 Department of Otolaryngology—Head and Neck Surgery, University of Texas-Southwestern, Dallas, TX, USA

**Keywords:** superior laryngeal nerve block, chronic cough, neurogenic cough, muscle tension dysphonia, paralaryngeal pain, inducible laryngeal obstruction, globus

## Abstract

**Objective:**

Superior laryngeal nerve (SLN) block consists of injection of steroid and anesthetic at the internal branch of the SLN entry site. Prior case series have demonstrated beneficial effects on neurogenic cough. SLN blocks have also recently shown benefit for paralaryngeal pain. We describe short-term outcomes for multiple symptoms of irritable larynx syndrome (ILS) including neurogenic cough, dysphonia related to laryngeal hypersensitivity, inducible laryngeal obstruction (ILO), paralaryngeal pain, and isolated globus.

**Methods:**

Retrospective review from 2 institutions of patients undergoing a single SLN block for the indications listed. Variables include age, sex, indication(s), known vagus neuropathy, and patient-reported outcomes at short-term follow-up.

**Results:**

A total of 209 patients were included (59 males, 150 females; age: 58 ± 13 years). Twenty-six patients (12%) had a history of a vagus nerve injury. Indications included neurogenic cough (n = 149), dysphonia related to laryngeal hypersensitivity (n = 66), paralaryngeal pain (n = 50), ILO (n = 23), and isolated globus (n = 3). Some patients had multiple indications. Significant improvements in patient-reported measures occurred after a single SLN block within 2 to 4 weeks for neurogenic cough (cough severity index; 25.2 ± 11.2 to 19.0 ± 12.8; *P* < .001), dysphonia (voice handicap index-10; 22.1 ± 12.2-18.0 ± 13.3; *P* = .005), and ILO (dyspnea index; 21.0 ± 14.9-14.7 ± 15.7; *P* = .017). Subjective pain improved in 23 of 39 patients with paralaryngeal pain. There was no observed improvement for isolated globus. Presence of known vagal neuropathy or therapy around the time of SLN block did not affect outcome.

**Conclusion:**

SLN block can be an effective component of treatment for a variety of ILS symptoms. Patients may experience some improvement after 1 injection.

**Lay Summary:**

Symptoms of irritable larynx syndrome, such as neurogenic cough, paralaryngeal pain, inducible laryngeal obstruction, and dysphonia related to laryngeal hypersensitivity can be challenging to manage. In-office Superior Laryngeal Nerve blocks can serve as a quick, well tolerated, adjunctive treatment with positive short-term outcomes.

**Level of evidence:**

4

## Introduction

Cough is one of the most common presenting complaints in the United States.^1^ Chronic neurogenic cough persists more than 8 weeks and is a diagnosis of exclusion after a negative workup for medication effect, pulmonary etiologies, gastroesophageal reflux disease (GERD), allergic rhinitis, or upper airway cough syndrome. Diagnosis and management of neurogenic cough is particularly challenging for otolaryngologists given nonspecific accompanying symptoms (ie, persistent throat clearing), inconsistent triggers for cough, and symptoms which often overlap with other laryngeal pathologies.^2^ Chronic cough often exists as a manifestation of irritable larynx syndrome (ILS), which can encompass a range of symptoms including throat clearing, throat hypersensitivity, globus pharyngeus, dysphonia, and inducible laryngeal obstruction (ILO).^3,4^ Cough can be particularly debilitating and a source of significantly diminished quality of life.^5^ To compound this, in the COVID-era, persistent coughing is stigmatized in a public setting.^6^

Current employed treatment options for neurogenic cough and ILS include behavioral cough suppression therapy (BCST) and systemic neuromodulator medications (eg, amitriptyline and GABA analogs).^7,8^ It can be difficult for some patients to access speech-language pathologists with the necessary expertise to provide this therapy, and the sedating effects of neuromodulators can be prohibitive.

One potential alternative or adjunctive approach to treating neurogenic cough involves addressing the affected sensory nerve directly. Simpson et al. proposed the Superior Laryngeal Nerve (SLN) block in 2018, which involves injection of a long-acting local anesthetic with steroid mixture targeted at the entry site for the internal branch of the SLN.^9^ The procedure is performed in the clinic setting with the patient seated upright, takes less than 1 minute to perform, and is well tolerated with a low complication rate.^9^ In the short term, a single injection may sometimes be sufficient, or the injection can be repeated for persistent or recurrent symptoms. Similar to accepted mechanism of neuromodulators used to treat neurogenic cough, SLN is hypothesized to alter sensory feedback and disrupt the cough signaling pathway.^9^ Further, cough and pain share a common receptor, TRPV1, and temporary peripheral nerve block may suppress nociceptive discharge and disrupt an aberrant sensory feedback loop promoting cough.^10^ In our initial study, 23 patients with neurogenic cough underwent an average of 2.4 injections with mean follow-up of 85 days and reduction in mean cough severity index score from 26.8 to 14.6.^9^

Since that initial study, other groups have reported their experience and noted similar benefits. Duffy et al. demonstrated a change in mean cough severity index score from 24.3 to 16.15 in 20 patients at short-term follow-up.^11^ Dhillon et al. performed an average of 3 injections in 30 patients with mean follow-up of 5.3 months and reduction in mean cough severity index score from 27 to 11.^12,13^ Larger studies with more patients would be helpful to further guide patient counseling on expected probability of improvement after an injection.

If SLN block can be helpful for treatment of neurogenic cough, it may potentially be helpful for other symptoms of ILS and superior laryngeal sensory neuropathy as well. As a first step, we recently demonstrated an improvement in paralaryngeal pain in 23 of 28 patients undergoing an average of 2.5 injections.^14^

The objective of this multi-institutional study is to add to the body of literature on SLN block outcomes for treating neurogenic cough and to evaluate the effect of the SLN block on other ILS symptoms, including dysphonia related to laryngeal hypersensitivity, paralaryngeal pain, ILO, and isolated globus pharyngeus. We hypothesized that SLN block would provide symptomatic benefit across several ILS symptoms at short-term follow-up.

## Methods

### Patients

This was a retrospective review approved by the University of Texas Health San Antonio and University of Alabama-Birmingham institutional review boards. All patients undergoing SLN block were eligible for inclusion. Records were reviewed from November 2015 to November 2020 at the University of Texas Health San Antonio and from March 2020 to August 2021 at the University of Alabama-Birmingham. Charts were reviewed for demographic information, indication for SLN block, history of vagus neuropathy, history of therapy with speech-language pathologist, and scores on patient-reported outcome measures. Indications for SLN block evaluated in this study included neurogenic cough, dysphonia related to laryngeal hypersensitivity, paralaryngeal pain, ILO, and isolated globus pharyngeus. Patients were excluded from the study if they did not have one of the above diagnoses.

Speech-langue pathologist therapy is a cornerstone of the management of the above conditions. In some cases of neurogenic cough or paralaryngeal pain, the SLN block may serve as an isolated, short-term treatment. In most cases, particularly for persistent neurogenic cough, dysphonia related to laryngeal hypersensitivity, and ILO, the SLN block serves as an adjunct to therapy and potential pharmacologic treatment. This study evaluated the short-term effect of a single SLN block on various ILS symptoms to determine which may respond to this intervention. The focus of this study is only on short-term outcomes (ie, 2- to 4-week follow-up after single injection). Participation in therapy around the time of injection was included as a variable as described in the statistical analysis.

### Procedural Interventions

Patients underwent SLN block as described previously by Simpson et al.^9^ A 1:1 mixture of triamcinolone (40 mg/ml) and 0.5% bupivacaine, total of 2 cc, is placed into a 3-cc syringe with 27-G needle. The thyrohyoid membrane is palpated, and the entry point of the superior laryngeal nerve is identified. The solution is then injected after drawing back on the syringe to ensure there is no intravascular injection. For patients with paralaryngeal pain, 1 cc of the mixture was deposited at the superior laryngeal nerve entry site and the other 1 cc was deposited at the posterior border of the thyroid cartilage.

### Questionnaire Responses

Patients typically completed 3 validated symptom indices: Cough Severity Index (CSI), Voice Handicap Index-10 (VHI-10), and a Dyspnea Index (DI). The CSI is a 10-item questionnaire that quantifies symptoms of cough with an upper airway origin.^15^ The VHI-10 is a short form of the Voice Handicap Index^16^ that describes the impact of dysphonia on quality of life.^17^ The DI is a 10-item questionnaire that captures upper airway-related dyspnea symptoms.^18^

Symptom indices were completed by the patient at the first visit prior to the SLN block and then at the initial follow-up post-injection visit, approximately 2 to 4 weeks afterward. All patients undergoing SLN block were recommended for BCST with a speech-language pathologist and the majority of those undergoing therapy pursued additional adjuvant SLN blocks. The focus of this study was on the short-term change occurring after a single injection.

### Statistical Analysis

Patients were separated into groups according to diagnosis. Comorbid diagnoses were considered as additional independent variables in analysis. Pre- and post-treatment values for CSI, VHI-10, and DI were compared using paired *t*-tests. If data did not meet assumptions for parametric testing, Wilcoxon signed rank tests were performed. Significance level for multiple paired analyses within a given diagnosis category was adjusted using the Bonferroni correction (α = .05/3 = .0167). Only patients with both pre- and post-treatment data were included in the paired analyses.

Analysis of covariance (ANCOVA) was used to evaluate potential factors affecting change patient-reported outcome measures. Independent variables included age, sex, presence of vagus nerve neuropathy, comorbid diagnoses, and participation in therapy within preceding 3 months prior to SLN block or between SLN block and post-SLN block follow-up visit. A window prior to the block was included as patients may improve carryover of techniques learned in therapy and thus could experience ongoing symptom improvement unrelated to the block. For this specific sub-analysis on effect of therapy, patients who underwent therapy with all sessions being more than 3 months prior to the SLN block were excluded. They were still included in analysis of other variables within the ANCOVA. For neurogenic cough, the primary measure of interest was the CSI. For dysphonia related to laryngeal hypersensitivity, the primary measure of interest was the VHI-10. For ILO, the primary measure of interest was the DI. Only patients with both pre- and post-treatment data were included in the ANCOVA analyses.

For paralaryngeal pain and isolated globus pharyngeus, the main outcome of interest was a subjective report of overall improvement or lack of improvement in symptoms as specified in the clinic notes. A binary logistic regression was performed using overall outcome (improvement or no improvement) as the binary dependent variable and the independent variables of age, sex, presence of vagus neuropathy, and comorbid diagnoses. Patients without a specified outcome in clinical documentation were excluded in the logistic regression analyses.

A significance level of α = .05 was used for the ANCOVA and logistic regression analyses. Univariate analyses were performed with subsequent multivariate analyses performed if multiple variables were found to be significant on univariate analysis. If 0 or 1 variables were significant on univariate analysis, a multivariate analysis was not performed. Data for each subgroup are presented descriptively. SPSS was used for analyses (IBM, Armonk, NY).

## Results

### Summary Data

**Figure 1.**
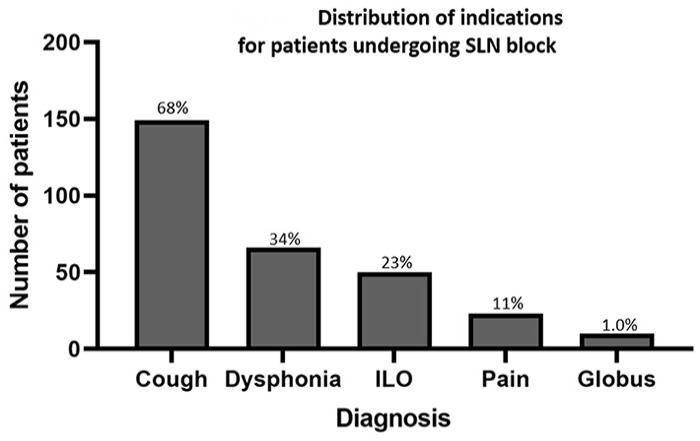
Distribution of primary indications for patients undergoing superior laryngeal nerve block. % at top of bar = relative proportion of total patients. Abbreviation: ILO, inducible laryngeal obstruction.

Summary data are presented as mean ± standard deviation unless stated otherwise. A total of 209 patients were included (59 men, 150 women) with age of 58 ± 13 years (Table 1). Twenty-six patients (12%) had a history of a vagus nerve injury. There were 114 (52%) patients who underwent at least 1 session of BCST prior to injection. Indications for SLN block included neurogenic cough (n = 149), therapy refractory MTD (n = 66), paralaryngeal pain (n = 50), ILO (n = 23), and isolated globus pharyngeus (n = 3; Figure 1). Some patients had more than 1 diagnosis prompting the procedure.

**Table 1.** Population Demographics and SLN Block Diagnoses.

Variable	All (N = 218)
Gender, n (%)	
Male	61 (28)
Female	157 (72)
Median age (range)	59 (25-88)
SLN block indication^a^, n (%)	
Neurogenic cough	149 (68)
Dysphonia	75 (34)
Paralaryngeal pain	50 (23)
ILO	23 (11)
Isolated globus	3 (1.0)

a Several patients had overlapping indications.

SLN = superior laryngeal nerve; ILO = inducible laryngeal obstruction.

### Neurogenic cough

**Figure 2.**
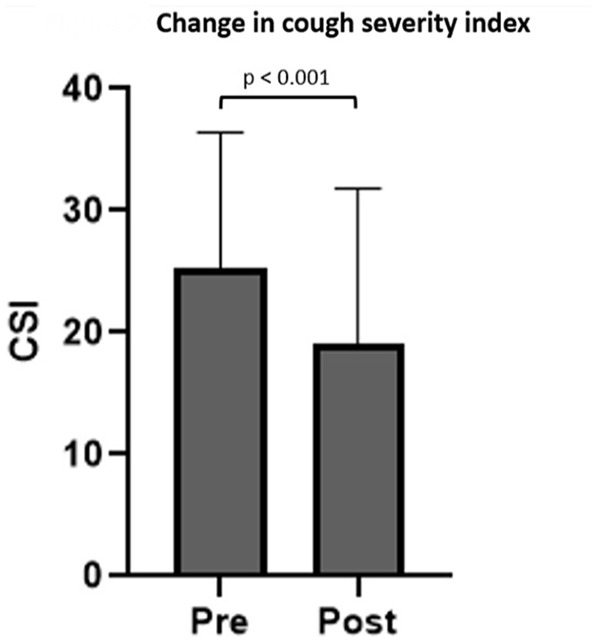
Bar graph depicting pre- and posttreatment cough severity index after a single superior laryngeal nerve block in patients with neurogenic cough. Bar height represents mean and error bars represent standard deviation. Abbreviation: CSI, cough severity index.

Neurogenic cough was the most common indication for SLN block (149/218, 68%). Pre- and post-treatment CSI scores were available for 83 patients, with 55 reporting improvement (66%), 12 reporting no change (14%), and 16 (19%) reporting higher score after a single injection. CSI scores decreased after SLN block (25.2 ± 11.2-19.0 ± 12.8; *t* = 5.386; 95% confidence interval [CI]: 3.9-8.5; *P* < .001; Figure 2). VHI-10 (*t* = 2.464; 95% CI: 0.5-4.3; *P* = .016) and DI (*t* = 3.450; 95% CI: 1.4-5.3; *P* < .001) also decreased in patients with neurogenic cough. Age, sex, history of vagus nerve injury, and comorbid diagnoses did not affect the change in CSI. Participation in therapy around time of SLN block also did not affect change in CSI at this short-term follow-up point (*F* = 0.605; *P* = .440).

### Dysphonia Related to Laryngeal Hypersensitivity

**Figure 3.**
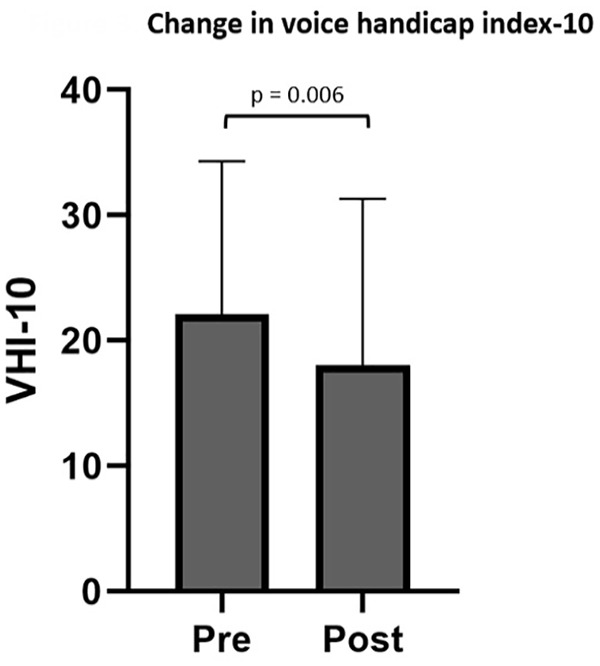
Bar graph depicting pre- and posttreatment voice handicap index-10 after a single superior laryngeal nerve block in patients with muscle tension dysphonia. Bar height represents mean and error bars represent standard deviation. Abbreviation: VHI-10, Voice Handicap Index-10.

Dysphonia was present in 66 of 209 patients (32%). Of the 66 patients, 47 had comorbid cough, 20 had paralaryngeal pain, 4 had ILO, and 2 had globus pharyngeus. Pre- and post-treatment VHI-10 scores were available for 37 patients, with 18 (49%) reporting improvement, 7 (19%) reporting no change, and 12 (32%) reporting higher score after a single injection. There was a reduction in VHI-10 score after SLN block (22.1 ± 12.2-18.0 ± 13.3; *t* = 2.961; *P* = .005; 95% CI: 1.3-6.9) (Figure 3) as well as CSI (*t* = 2.762; 95% CI: 1.3-8.7; *P* = .009) and DI (*t* = 2.696; 95% CI: 0.8-5.7; *P* = .01). Age, sex, history of vagus nerve injury, and comorbid cough, ILO, paralaryngeal pain, or globus did not affect the change in VHI-10 score. All patients underwent therapy with a speech-language pathologist as part of their treatment. Participation in therapy around time of SLN block did not affect change in VHI-10 score at the short-term follow-up point (*F* = 2.384; *P* = .132).

### Paralaryngeal Pain

Paralaryngeal pain was present in 50/218 patients (23%). For patients with explicitly reported comment on improvement, 23 of 39 patients experienced improvement (59%). Age, sex, and comorbid cough or MTD did not have an impact on likelihood of improvement. Of these 50 patients, 28 underwent therapy with a speech-language pathologist as part of their treatment.

### Inducible Laryngeal Obstruction

**Figure 4.**
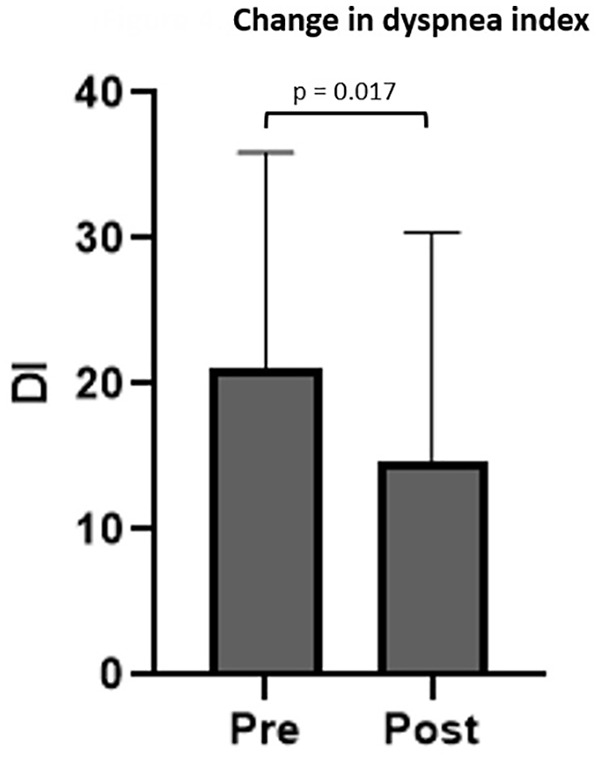
Bar graph depicting pre- and posttreatment dyspnea index after a single superior laryngeal nerve block in patients with inducible laryngeal obstruction. Bar height represents mean and error bars represent standard deviation. Abbreviation: DI, Dyspnea Index.

Inducible laryngeal obstruction was present in 23 of 218 patients (11%). Pre- and post-treatment DI scores were available for 10 patients, 7 (70%) reporting improvement and 3 (30%) reporting no change after a single injection. No patients reported worsening. DI score decreased from 21.0 ± 14.9 to 14.7 ± 15.7 (*t* = 2.926; 95% CI: 2.3-18.1; *P* = .017; Figure 4). Age, sex, history of vagus nerve injury, and comorbid cough or MTD did not have an impact on the change in DI. Of 23 patients, 15 underwent therapy with a speech-language pathologist as part of their treatment. Participation in therapy around time of SLN block also did not affect change in DI score at this short-term follow-up point (*F* = 0.049; *P* = .834).

### Isolated Globus Pharyngeus

Isolated globus pharyngeus (foreign body sensation as the only presenting symptom) was present in 3/218 patients (1%). Presence or absence of improvement was explicitly documented and none of the 3 patients with isolated globus pharyngeus improved. Age or sex did not have an impact on likelihood of improvement.

### Complications

There were 2 documented adverse reactions: self-limiting ecchymosis at the injection site and temporary tongue paresthesia.

## Discussion

This is the largest study to date evaluating outcomes of SLN block among patients with neurogenic cough and expands on the role of SLN block to other symptoms associated with ILS. After 1 injection, most patients reported overall improvement in symptoms of neurogenic cough, dysphonia related to laryngeal hypersensitivity, paralaryngeal pain, or ILO. There was no improvement with isolated globus pharyngeus. Patient reports of subjective improvement was supported by objective changes in relevant validated patient-reported outcome measures.

In general, the SLN block is best used as an adjunctive treatment, coupled with therapy administered by a speech-language pathologist. This is particularly true for longstanding neurogenic cough, dysphonia, and ILO. We have observed that some patients with dysphonia related to laryngeal hypersensitivity, particularly those with an element of odynophonia, can more readily participate in voice therapy after initial pain relief following an SLN block. Similarly, patients with refractory ILO may be more amendable to participate in and carryover the rescue breathing techniques learned in therapy after SLN block. In select cases when there is limited access to a speech-language pathologist with expertise managing symptoms of irritable larynx, SLN block may be considered as a primary treatment, often coupled with neuromodulator therapy. In this dataset, participation in therapy around the time of SLN block did not affect the magnitude of improvement in the patient-reported outcome measures. This is likely related to the short-term follow-up period which was used. Longer-term follow-up is more likely to show the synergistic benefits of multimodal therapy. As shown in the data, there was improvement in symptoms but not resolution of symptoms after a single SLN block at short-term follow-up. This emphasizes the need for multimodal therapy and longer treatment course. These data are helpful for patient counseling on expectations for improvement following a single injection.

We found that approximately 3 of 4 patients who underwent SLN blocks were women. A significant female predominance is in accordance with prior studies on ILS noting a female:male ratio of 2 to 3:1.^19-21^ Also in congruence with prior studies, injections were well tolerated in nearly all patients in this study with only 2 documented and temporary complications.

A subset of our cohort (26 patients) had known vagal nerve injury. Anecdotally, we have observed that patients with history of vagal nerve injury and either neurogenic cough or ILO seem to respond favorably to SLN block. This may be in line with what was observed by Duffy et al., where patients with asymmetric motion/vibratory finding on laryngoscopy had higher likelihood of improvement following the procedure.^11^ Patients with either iatrogenic or post-viral vagal nerve branch injury may report cough, throat tickle, or persistent throat clearing.^19^ After reviewing the patient-reported outcome measures in this larger dataset, though, we did not observe a significant impact of vagal nerve injury on outcomes. Most patients, regardless of presence or absence of vagal nerve injury history, tended to improve in this study. The potential relationship between history of vagal nerve branch injury and response to SLN block will be evaluated in future studies.

Findings from this study in patients with neurogenic cough parallel our initial study as well as those from Duffy et al^11^ and Dhillon^9,12,13^ When counseling patients, at least short-term improvement in cough-related symptoms can be expected to occur in the majority of patients. Most patient reported improvement in cough and had significant reduction in CSI pre- and post-treatment scores.

This study expands on the potential roles of the SLN block by demonstrating improvement in dysphonia related to laryngeal hypersensitivity, paralaryngeal pain, and ILO. The recent publication by Tibbets et al. supports use of the SLN block for paralaryngeal pain with 82% of patients reporting improvement.^14^ This was higher than that observed in our study, with 59% reporting improvement. This can likely be attributed to the number of injections performed. We have observed clinically that patients with paralaryngeal pain often requires serial injections over a period of 1 to 2 months to achieve optimal pain relief. In this study, we focused on the short-term effects of a single injection. Given that these patients often present with a long history of bothersome pain, the finding that 59% of patients experienced benefit after a single injection is encouraging.

The SLN block is simple to perform, low-cost, and requires common equipment available in any otolaryngology office. Additionally, complication rate is low and tolerance is high. It offers an alternative or adjunctive option for patients who may have difficulty tolerating or incomplete benefit from neuromodulators. Similarly, it can be offered to patients who do not have complete symptom resolution following therapy with a speech-language pathologist or who may have a delay prior to participating in therapy. One notable limitation of the SLN block is that patients may require repeated injections with alternating laterality given the variable response and duration of therapeutic effect. This study provides clinicians with summary results that can be used to counsel patients who appropriately ask what impact on symptoms can be expected from a single injection.

Patient selection is critical to maximizing probability of improvement following an SLN block. A clinician must ensure an accurate diagnosis and then determine laterality when applicable. The patient population with neurogenic cough is distinct and in most cases, the primary causes of chronic cough have been ruled out prior to treatment. This typically includes pulmonary function testing and chest imaging, a trial of high dose proton pump inhibitor (PPI) therapy, and empiric treatment (including daily nasal corticosteroids) for upper airway cough syndrome for at least 4 weeks. Many patients had also undergone esophagogastroduodenoscopy prior to SLN block to rule out possible GERD as a primary cause of cough. It is important to rule out these common causes of cough and perform flexible laryngoscopy to rule out underlying structural or pathologic abnormality which may be prompting cough prior to diagnosing a patient with neurogenic cough and considering SLN block as a treatment option.

The data from this study suggest that there is no symptomatic improvement when performing SLN block for isolated globus pharyngeus, although this was the smallest cohort in our study. Globus sensation is a nonspecific symptom with numerous potential causes. Additionally, globus pharyngeus does not frequently have a laterality, and so response to a single SLN block may be less predictable. In this study, SLN was attempted as patients had already tried traditional therapies such as PPI for possible GERD, nasal steroids for possible allergic rhinitis, and hydration-focused measures for possible laryngitis sicca. SLN block is a potential low-risk and low-cost option, but the limited data available do not show reliable benefit for globus pharyngeus.

Several limitations should be considered. This was a retrospective cohort study without an available group for comparison and the study was not designed to be prospective with a control group for comparison. Therefore, a placebo effect could certainly have an impact on the patient-reported outcome measures. Additionally, patient medical comorbidities ongoing at the time of SLN block or post-injection data capture were not accurately charted which can also contribute to subjective changes in cough or dyspnea; hence, this was unable to be controlled for in analysis to isolate the effect of the SLN block alone. The mechanism of how SLN injection affects the nerve at the physiologic level is theorized but not understood. Further, this study focused only on the short-term outcome of a single injection for multiple indications. Longer-term outcomes were not the focus of this study. The study was performed at 2 institutions, with potential for some heterogeneity in how the procedure was performed. However, given the simple nature of the procedure, the multi-institutional nature primarily serves to improve study result generalizability. Lastly, only subjective patient-reported outcome measures were available, and no quantitative cough or voice analyses were performed. Future investigations will include prospective placebo-controlled investigations with longer-term follow-up and multi-parametric outcome assessment.

## Conclusion

Office-based SLN block can decrease symptom burden for multiple aspects of ILS, including neurogenic cough, dysphonia related to laryngeal hypersensitivity, paralaryngeal pain, and ILO. There does not appear to be a benefit for patients with isolated globus pharyngeus.
